# Circlehunter: a tool to identify extrachromosomal circular DNA from ATAC-Seq data

**DOI:** 10.1038/s41389-023-00476-0

**Published:** 2023-05-22

**Authors:** Manqiu Yang, Shufan Zhang, Rong Jiang, Shaomu Chen, Moli Huang

**Affiliations:** 1 School of Biology and Basic Medical Sciences, Soochow University, 215123 Suzhou, China; 2 Department of Thoracic Surgery, The First Affiliated Hospital of Soochow University, 215006 Suzhou, China

**Keywords:** Cancer genomics, Small-cell lung cancer, Gene amplification

## Abstract

In cancer, extrachromosomal circular DNA (ecDNA), or megabase-pair amplified circular DNA, plays an essential role in intercellular heterogeneity and tumor cell revolution because of its non-Mendelian inheritance. We developed circlehunter (https://github.com/suda-huanglab/circlehunter), a tool for identifying ecDNA from ATAC-Seq data using the enhanced chromatin accessibility of ecDNA. Using simulated data, we showed that circlehunter has an F1 score of 0.93 at 30× local depth and read lengths as short as 35 bp. Based on 1312 ecDNAs predicted from 94 publicly available datasets of ATAC-Seq assays, we found 37 oncogenes contained in these ecDNAs with amplification characteristics. In small cell lung cancer cell lines, ecDNA containing *MYC* leads to amplification of *MYC* and cis-regulates the expression of *NEUROD1*, resulting in an expression pattern consistent with the *NEUROD1* high expression subtype and sensitive to Aurora kinase inhibitors. This showcases that circlehunter could serve as a valuable pipeline for the investigation of tumorigenesis.

## Introduction

Extrachromosomal circular DNA (ecDNA) is circular DNA molecules outside the chromosome [1, 2]. In contrast to microDNA, the common, seemingly small, and gene-free, consisting primarily of 200–500 bp repetitive DNA fragments found in eukaryotes. ecDNA is typically found only in cancer samples, typically has a size over 1 million base pairs, and contains both genes and noncoding DNA, including regulatory regions [2–7]. Since it was first reported in 1965 [8], an increasing number of ecDNAs have been found to carry important oncogenes in different types of cancer [9], including *MYC*, *MYCN*, and *EGFR* [10–12]. Since ecDNA lacks centromeres, they may segregate randomly during mitosis, and the resulting amplification of oncogenes enables cancer cells to rapidly acquire evolutionary fitness and drug resistance [1, 3, 13]. In addition to the high copy number due to gene amplification, the increased accessibility due to reduced nucleosome wrapping, and the proximity of cis-regulatory elements due to circularization, increased the expression level of oncogenes on ecDNA to the top 1% in the whole transcriptome and contributed to the pathogenesis of tumors [4–6]. Currently, there are several strategies for identifying ecDNA on a whole-genome scale based on sequencing technologies. AmpliconArchitect is an ecDNA identification framework based on whole-genome sequencing (WGS) technology that constructs ecDNA by analyzing the junction relationships between amplified fragments [14]. This approach recommends 5–10× WGS data [14], which involves many computational resources. An alternative approach is constructing sequencing libraries by digesting linear DNA using exonucleases and enriching circular DNA using a rolling circle amplification (RCA) technique to generate sequencing libraries [15]. However, the technical challenges of this approach remain, especially for ecDNA of several megabase pairs long, where the avoidance of circular DNA damage and high-fidelity amplification are critical for success [1]. Recently, the Circle_finder method, which uses assay for transposase-accessible chromatin using sequencing (ATAC-Seq) data to discover junctions by collecting supplementary alignment reads, which has been reported to identify small circular DNAs or even ecDNAs [16]. However, given the stringent conditions of the supplementary alignment, which limited the number of reads to prove a junction (Supplementary Fig. S1B), particularly the lack of consideration for continuity between fragments and complex rearrangements, the method cannot detect the complex ecDNA (Supplementary Fig. S1A). ATAC-Seq is a well-established technique for assessing chromosomal accessibility, using the Tn5 transposase to insert sequencing adapters into open regions of chromosomes to construct a sequencing library [17]. It has been shown that ecDNA has less higher-order chromatin compaction compared to DNA on chromosomes and offers significantly enhanced chromatin accessibility, distinguishing them from linear duplication [6, 9]. Analysis of known ecDNA shows that the ATAC-Seq signals reach a minimum local depth of 80× for ecDNA at an overall sequencing depth of 1–3× (Supplementary Table S2). Therefore, we developed circlehunter to reconstruct ecDNA by taking full advantage of the increased ATAC-Seq signals arising from the high accessibility of ecDNA itself. By taking advantage of this feature of ecDNA, we can avoid the RCA step and still enrich for ecDNA sequence. Using simulated data, we demonstrate that circlehunter still has high accuracy for short read lengths and low depth. Using circlehunter, 1312 ecDNA were identified in 94 publicly available historical data of tumor samples, providing a new perspective for studying the oncogenicity of ecDNA.

## Results

### Principle of identification method

**Fig. 1:**
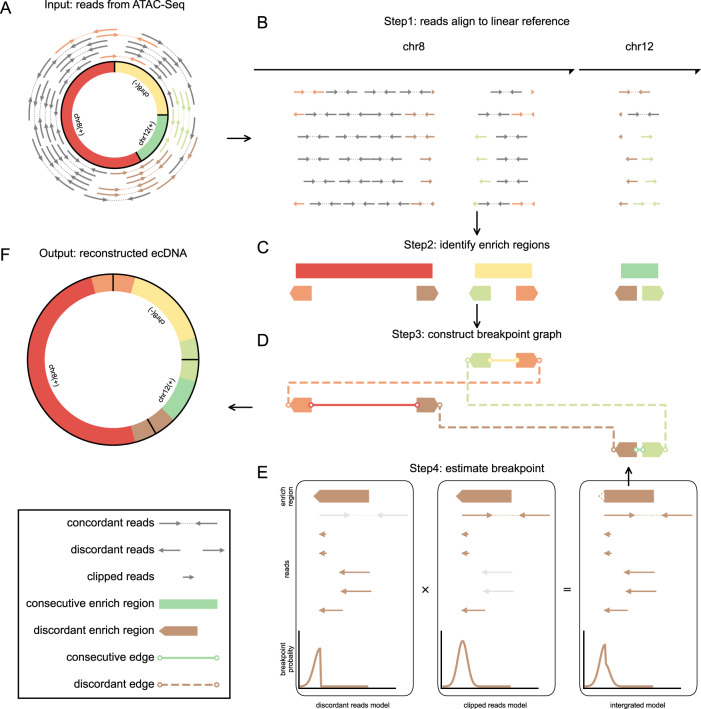
Principle of identification method. **A** Lack of nucleosome-packed ecDNA generates a large number of reads in ATAC-Seq. **B** Reads mapped to genome reference. **C** Identify consecutive enrich regions and discordant read pair enrich regions. **D** Breakpoints graph linked by discordant read pair enrichment and consecutive read enrichment. **E** Bayesian models estimate breakpoints according to discordant read pairs (left) and clipped reads (middle). **F** Reconstruct ecDNA from the breakpoint graph.

Extensive research has shown that ecDNA has a chromosomal origin [1, 18]. DNA damage, such as chromothripsis, breaks chromosomes into small pieces. These linear DNA fragments can be relegated to head-to-tail orientation due to the DNA repair mechanism to form an ecDNA [19–22]. It has been shown that ecDNA has much more open chromatin and shows significantly enhanced chromatin accessibility [6, 9]. These results imply that the source regions of ecDNA would exhibit consecutive reads enrichment in ATAC-Seq sequencing results. Thus, the identification method can be processed in the following steps. First, the large number of reads from ecDNA segments generated due to the lack of nucleosome packing can be aligned to their chromosomal origin (Fig. 1A, B). Segments that may form an ecDNA can be identified as consecutive reads enrichment (Fig. 1C). Discordant reads pair enrich region may link with another segment (Fig. 1C) and indicate a candidate region contain a breakpoint. So, we can construct a breakpoints graph linked by discordant reads pairs and consecutive enrichment (Fig. 1D). All possible circular DNA can be searched from the breakpoint graph (Fig. 1F). To determine the exact breakpoint from the discordant reads pair enriched region but avoid time-consuming realignment, an integrated Bayesian model is used to estimate the precise breakpoints of fragments (Fig. 1E). The detailed workflow is described in “Materials and methods”. In contrast to the existing circle_finder method, the reads used by circlehunter as evidence cover the entire length of the predicted ecDNA, enabling circlehunter to identify segments that are ligated circularly and discover genetic variants resulting from the ligation (Supplementary Fig. S1A).

### Accuracy of circlehunter

**Fig. 2:**
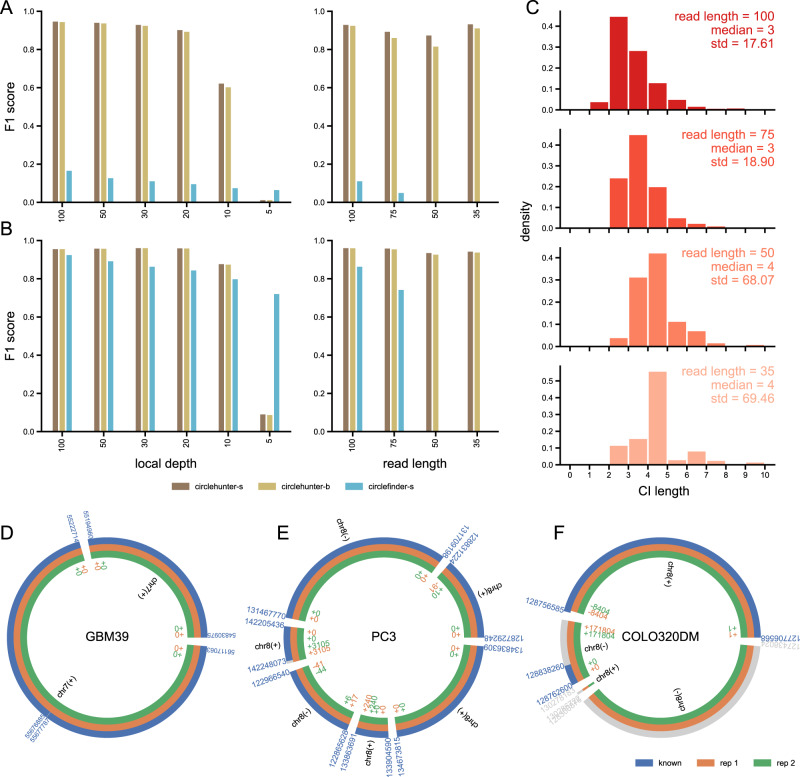
Accuracy of circlehunter. **A** F1 score of multi segments ecDNA detection with varying mock ecDNA local depth at 100 bp read length and varying read length at 30× local depth. **B** F1 score of single-segment ecDNA detection with varying mock ecDNA local depth at 100 bp read length and varying read length at 30× local depth. Circlehunter-s: A true-positive result is considered when the circlehunter result covers 95% of the simulated ecDNA and has the same ligation structure; Circlehunter-b: A true-positive result is considered when the circlehunter result breakpoint confidential intervals cover simulated ecDNA breakpoint and have the same ligation structure; Circlefinder-s: A true-positive result is considered when the Circle_finder result covers 95% of the simulated ecDNA. **C** Distribution of 95% confidential interval length with varying read length at 30× local depth. **D**–**F** Identify results of GBM39, PC3, and COLO320DM cell lines. Blue is the validated result, and orange and green are the individual results of two biological replicates. The color of the numbers indicating the genomic position corresponds to the circles. The numbers outside the circles are the absolute positions of the validated ecDNA fragments on the genome. The numbers inside the circles are the errors of the ecDNA fragments identified by circlehunter relative to the validated positions.

To examine the ability of circlehunter to identify circular DNA from the linear genome reference, we tested it using 500 simulated ecDNA randomly selecting chromosomal regions of the human genome. These mock ecDNAs’ length ranges from 5 kb to 10 Mb, and the number of segments ranges from 1 to 50, covering various conditions of ecDNA (Supplementary Fig. S1C, D). For short reads sequencing, analysis of long structural variants is usually limited by the coverage and read length. We simulated different ecDNA local depth and read-length datasets to check the detection performance of circlehunter under different conditions. We also compared it with the existing tool Circle_finder, which can analyze ecDNA from ATAC-Seq [16]. The test result showed that circlehunter could reconstruct the most mock ecDNA in different local depth datasets. Since circlehunter only considers segments linked with significant number of reads, it has higher precision and recall than circle_finder when local depth is less than 10× (Supplementary Fig. S1E–H and Supplementary Table S1). For comparison purposes, we introduce the F1 score to evaluate the detection performance of each tool. The results shown that circlehunter outperforms circle_finder at different local depth levels except with local depth less than 10× (Fig. 2A, left), with an F1 score of 0.93 at local depth greater than 30×. On this basis, we test the effect of varying read length on the detection performance at the local depth of 30×. The results showed that circlehunter was barely affected by read length, but circle_finder could not obtain any results when the read length was less than 75 bp (Fig. 2A, right). This is mainly because read length will significantly affect the appearance of supplementary alignment, but not the number of discordant reads pair. This enables circlehunter, which works on discordant read pairs, to be almost independent of read length. Still, the read length affects circlehunter’s estimate of the exact breakpoint, and a shorter read length will result in a larger confidence interval (Fig. 2C). Since circle_finder reports only those circular DNAs that are generated by end-to-end ligation of one linear fragment, we also generated a test set consisting only single-segment ecDNAs. The results show that circlehunter still outperforms circle_finder for single-segment ecDNA when the coverage is greater than 10× (Fig. 2B), although circle_finder has better detection performance for single-segment ecDNA test sets than for multi-segment ecDNA test sets (Fig. 2B). These results suggest that circlehunter can accurately detect ecDNA in samples when the local depth of ecDNA is sufficient (>30×). In comparison, the local depth of 11 ecDNAs segments from 6 replicates of 3 known samples was greater than 80× (Supplementary Table S2). At this point, the overall depth of the entire genome is between 1–3×, and the overall sequencing depth of most historical samples is also in this interval (Supplementary Fig. S2C) (Supplementary Table S3). In our collection of historical samples, the sequencing length is usually between 35 and 75 bp (Supplementary Fig. S2D) (Supplementary Table S3). All can be used as input data for circlehunter. As a negative control, we analyzed 14 ATAC-Seq datasets obtained from normal muscle tissue (Supplementary Table S1). Circlehunter only reported 1 ecDNA, demonstrating that it is unlikely to report non-amplified false positives. Also, circlehunter outperforms circle_finder in terms of runtime, memory usage, and IO throughput (Supplementary Fig. S1I–K) (Supplementary Table S1). These results indicated that circlehunter had high-performance detecting ecDNA and can be applied to most ATAC-Seq data.

Having observed that we can detect ecDNA from linear genomes in simulation samples, we tried to apply circlehunter to actual samples. Analysis of ATAC-Seq data from three samples with known ecDNA showed that circlehunter could identify ecDNA identified by WGS data and validated by fluorescence in situ hybridization (FISH) [6] (Fig. 2D–F) (Supplementary Table S2). The ecDNA in cell line GBM39 has a simple composition, consisting of a long contiguous fragment joined end-to-end with certain deletions in between. Besides minor deletions, circlehunter can accurately identify all breakpoints and their linkage relationships (Fig. 2D). In contrast, the known ecDNA in the PC3 cell line consists of six fragments joined in multiple ligation directions. However, circlehunter still accurately identifies all ligation relationships and predicts most breakpoints (Fig. 2E). The most challenging trial was identifying the ecDNA present in COLO320DM, a typical highly chromosomal rearrangement cell line where the ecDNA present may have complex linkage relationships with multiple possible ecDNA. Circlehunter did not identify the known ecDNA identically but identified ecDNA with a similar structure to the known ecDNA in two biological replicates (Fig. 2F). Based on the priority setting when searching for paths (see “Materials and methods”), we presume the result is an alternative ecDNA present in this sample or computational sub-structure. Overall, test results on both simulated and actual samples show that circlehunter has high accuracy and sensitivity in identifying ecDNA and is generally applicable to regular ATAC-Seq data.

### Application to public data

**Fig. 3:**
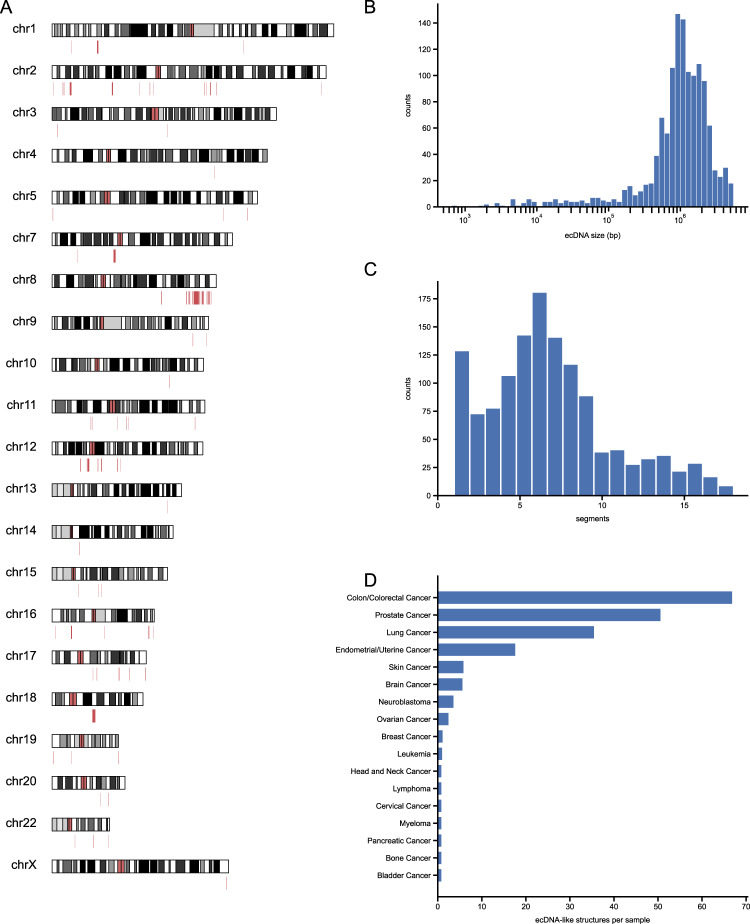
Statistics of ecDNAs. **A** Distribution of ecDNA on chromosomes. **B** Distribution of ecDNA size. *X* axis is log scaled. **C** Distribution of ecDNA segment counts. **D** Distribution of ecDNA-like structure in cancers.

Based on the high accuracy obtained from simulated and real data testing, we apply circlehunter to publicly available historical data. We curated ATAC-Seq for 547 tumor samples from patient tissues, patient-derived xenografts, and cancer cell lines (Supplementary Fig. S2A and Supplementary Table S3) from the Gene Expression Omnibus (GEO) database [23]. In total, 1312 candidate ecDNA (or microDNA) circular structures were predicted in 94 (17.18%) samples of 17 cancers (Supplementary Table S3) by circlehunter. These ecDNAs are sourced from all chromosomes except the Y chromosome (Fig. 3A) and range in size from 654 to 5,515,037 bp (Fig. 3B). Half of these ecDNAs are larger than 1 Mb in size (Fig. 3B), and 97.86% of these ecDNAs are larger than 10 kb, suggesting that these circles are mostly ecDNA-like. The median number of segments that constitute an ecDNA is 6, with a maximum of 18 segments (Fig. 3C). In terms of cancer types, the most ecDNA was identified in colorectal cancer, prostate cancer, and lung cancer (Fig. 3D), which have been shown to have recurrent circular amplification [9, 14, 24]. ecDNA was present in both primary and metastasis tumor samples, and there was no significant difference in the proportion of both (Supplementary Fig. S2E). Therefore, ecDNA may not be inevitably associated with tumor metastasis, but the amplification of genes within ecDNA may still impact tumor metastasis [25].

**Fig. 4:**
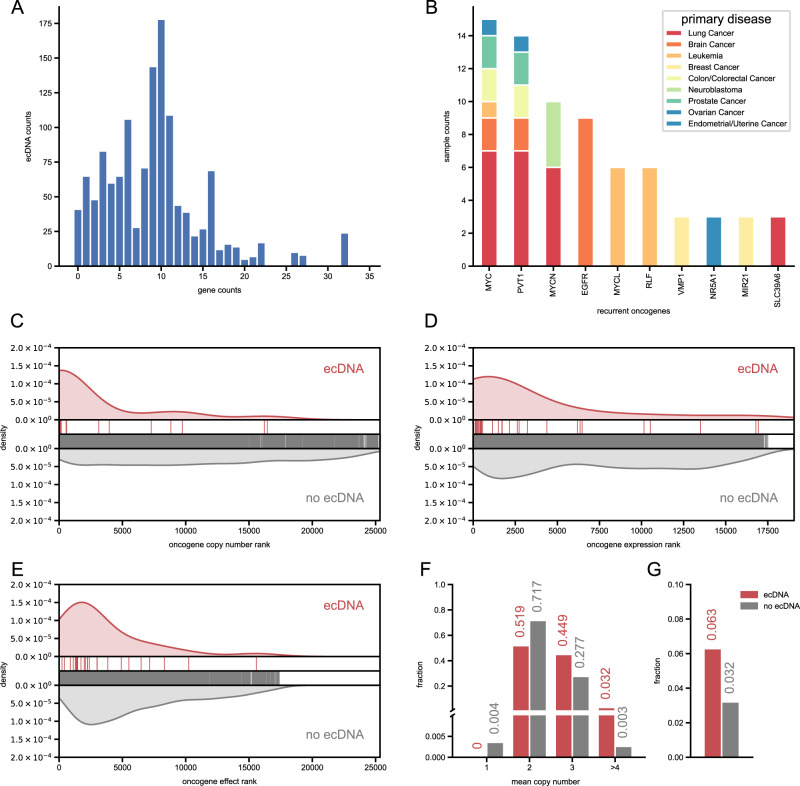
Genes contained in ecDNA. **A** Distribution of genes in ecDNA. **B** Distribution of recurrent oncogenes contained in ecDNA. **C** Kernel density estimate of copy number ascending rank between samples within ecDNA contained the oncogene, and those without ecDNA contained the oncogene. **D** Kernel density estimate of expression level ascending rank between samples within ecDNA contained the oncogene, and those without ecDNA contained the oncogene. **E** Kernel density estimate of gene effect rank between samples within ecDNA contained the oncogene, and those without ecDNA contained the oncogene. **F** Histogram of mean copy number for ecDNA-contained gene and gene never contained in any ecDNA. **G** The fraction of gene mutated in ecDNA-contained gene and gene never contained in any ecDNA.

There is variation in the number of genes contained within each ecDNA (Fig. 4A), with an average of 10 genes per ecDNA and a total of 251 genes amplified in all ecDNAs. Of these, 37 genes are known to be oncogenes. Certain oncogenes are commonly found on ecDNA and are associated with specific cancers. Three *myc* family genes, *MYC*, *MYCN*, and *MYCL*, are found in ecDNA in various cancers (Fig. 4B). However, *MYC* is usually amplified with *PVT1* (Fig. 4B). While *MYCN*-containing ecDNA is generally found in neuroblastoma, *MYCL*-containing ecDNA is only found in leukemia (Fig. 4B). *EGFR* is only amplified in brain cancer (Fig. 4B). Of the samples that have been analyzed, 109 cell lines have been characterized by the Cancer Cell Line Encyclopedia (CCLE) [26]. Using data from the CCLE, we confirmed that ecDNAs predicted by circlehunter have the characteristics of ecDNA, which are consistent with those observed in previous studies [9]. In ecDNA, genes typically have higher copy numbers and expression levels and affect cell survival (Supplementary Fig. S3A–C). At the same time, the copy number increase of highly expressed genes on ecDNA is correlated with the expression level increase, which is significantly different (*P* = 1.025 × 10^−3^, Fisher transformation z test) from the gene expression level increase driven by other means such as expression regulation (Supplementary Fig. S3D). These phenomena are more evident for oncogenes. For example, oncogenes in ecDNA had a significantly high copy number (*P* = 8.754 × 10^−16^, two-sided Wilcoxon rank-sum test) (Fig. 4C). Similarly, oncogenes contained in ecDNA had significantly higher expression levels at the transcriptional level (*P* = 2.047 × 10^−8^, two-sided Wilcoxon rank-sum test) (Fig. 4D). Furthermore, knockout of these genes identified as being contained within ecDNA had a significantly greater impact on the growth and survival of the corresponding cells compared to the other cell lines (*P* = 8.408 × 10^−5^, two-sided Wilcoxon rank-sum test) (Fig. 4E). These characteristics suggest that ecDNA may be an essential driver of cancer. This was confirmed in large-scale clinical data from the PanCancer Analysis of Whole Genomes (PCAWG) project, which showed that genes predicted to have circular amplification were also more likely to be coincident with regions of copy number gain (*P* = 1.619 × 10^−15^, Pearson’s chi-square test) (Fig. 4F). Similarly, these protein-coding genes, which are predicted to be contained within ecDNA, are also more likely to have somatic mutations (*P* = 4.986 × 10^−2^, Pearson’s chi-square test) (Fig. 4G). Taken together, these results show that ecDNAs predicted by circlehunter have the typical characteristics of known ecDNAs, which proves the detection performance of circlehunter from the side.

### ecDNA in small cell lung cancer (SCLC)

**Fig. 5:**
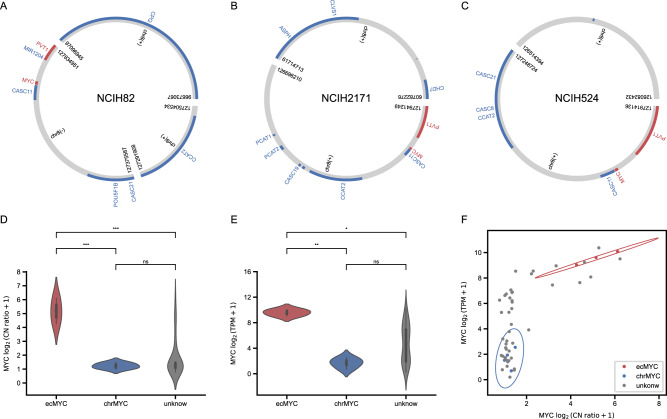
MYC amplified on ecDNA. **A**–**C** *MYC* contained ecDNA from NCI-H82, NCI-H2171, and NCI-H524 cell lines. **D** Violin plot of *MYC* copy number from SCLC cell lines. **E** *MYC* expression level from SCLC cell lines. Significant markers are ns for not significant, *P < 0.05, **P < 0.01, and ***P < 0.001. **F** Relationship between copy number and expression level of *MYC*. Confidence ellipses of ecMYC group (red) and chrMYC (blue) are shown, with confidence intervals represented by ellipses based on three times the standard deviation.

In the results of historical data analysis, we found that in SCLC, ecDNA containing *MYC* (ecMYC) was identified in several samples. SCLC is a highly aggressive subtype that accounts for about 15% of lung cancer cases with a dismal 5-year survival rate of about 6% [27]. Amplification of *myc* family genes has been shown to be a characteristic of SCLC [27–31]. A total of 19 ATAC-Seq data from 8 SCLC cell lines is included in the public sample set. We detected ecDNA in 4 SCLC cell lines (Supplementary Table S4). Specifically, ecDNA containing *MYCN* was present in NCI-69 (Supplementary Fig. S3E). In contrast, ecDNA containing *MYC* and *PVT1* was identified in NCI-H82 (Fig. 5A), NCI-H2171 (Fig. 5B), NCI-H524 (Fig. 5C), and notably, all these ecDNA fragments containing *PVT1*, their breakpoints fall into *PVT1* (Fig. 5A–C).

**Fig. 6:**
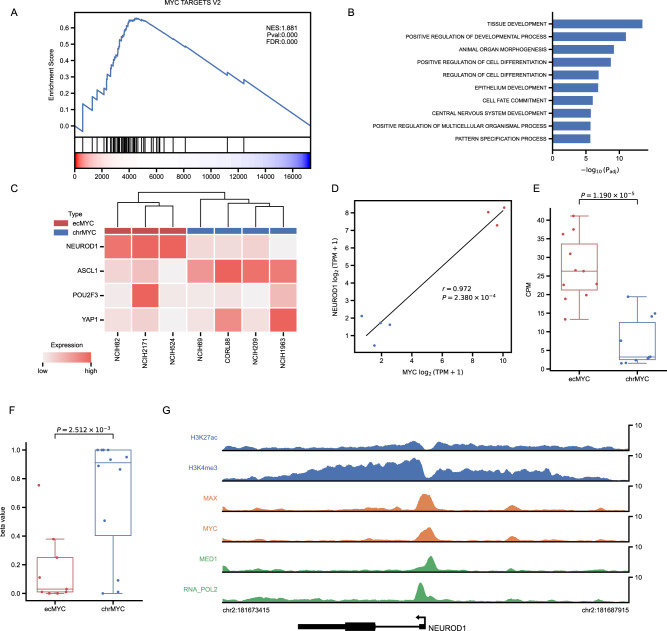
ecMYC-driven SCLC-N expression pattern. **A** GSEA result of term HALLMARK_MYC_TARGETS_V2 from MSigDB between SCLC cell lines with ecMYC and chrMYC. **B** GO analysis for differentially expressed genes between cell lines with ecMYC and chrMYC. **C** Heatmap of 4 SCLC subtype factors expression. **D** Correlation between *MYC* and *NEUROD1* expression levels in SCLC cell lines. **E** Accessibility of ecMYC and chrMYC cell lines at *NEUROD1* TSS. **F** The methylation level of the CpG cluster in the *NEUROD1* locus. **G** The epigenomic landscape of the NCIH-2171 cell line in *NEUROD1* locus.

We focused on examining the amplification of *MYC* on ecDNA. Copy number data from CCLE (NCI-H2107 not included) showed that cell lines with ecMYC had significantly higher copy numbers of the *MYC* than the rest of the SCLC cell lines (*P* = 1.425 × 10^−4^, ANOVA) (Fig. 5D). At the transcriptional level, *MYC* expression level was significantly higher than the rest of the SCLC cell lines (*P* = 2.327 × 10^−3^, ANOVA) (Fig. 5E), too. These cell lines were differentiated based on their *MYC* amplification status, with ecMYC exhibiting higher *MYC* copy number and expression levels (Fig. 5F). Gene Set Enrichment Analysis (GSEA) results showed that target genes of *MYC* were enriched in samples within ecMYC (Fig. 6A) (Supplementary Table S5), demonstrating that *MYC* was amplified on ecDNA in these samples and regulated the transcription level of *MYC* target genes as well. From the results of differentially expressed gene analysis, we obtained 321 genes whose expression levels were significantly different between cell lines with either ecMYC or *MYC* on the chromosome (chrMYC) (Supplementary Fig. S4A) (Supplementary Table S5). Gene Ontology (GO) analysis revealed that these genes were significantly enriched in pathways associated with developmental regulation and regulation of cell differentiation (Fig. 6B) (Supplementary Table S5), in agreement with the cancer hallmark of SCLC [27], suggesting that ecMYC may drive cells to develop specific expression patterns and lead to SCLC. Publicly available transcriptome sequencing data from 81 SCLC specimens indicate that this expression pattern is also observed in patients with high *MYC* expression who are enriched for differentially expressed genes obtained from cell lines (Supplementary Fig. S4B). *MYC* and *NEUROD1* were the most highly expressed genes (Supplementary Fig. S4A). Despite the paucity of data, we did observe a trend in which *MYC* and *NEUROD1* both affect the survival of cells with ecMYC (Supplementary Fig. S4C). Much of the research on SCLC has been conducted that molecular subtypes of SCLC are usually distinguished by the expression level of four subtype factors, including *NEUROD1*, rather than genomic mutations [32]. We, therefore, examined the expression of four SCLC subtype factors, and the high *NEUROD1* expression showed that these three cell lines with ecMYC all belong to the SCLC-N (*NEUROD1*) subtype (Fig. 6C). And the correlation between *MYC* and *NEUROD1* in terms of expression level further suggests a regulatory role of *MYC* on *NEUROD1* (Fig. 6D and Supplementary Fig. S4D). To examine the regulatory effects of ecMYC on *NEUROD1*, we first investigated the accessibility of the transcription start site (TSS) region of the *NEUROD1*, and the data showed that the accessibility of the TSS region of the *NEUROD1* was significantly higher in cell lines with ecMYC than chrMYC (Fig. 6E). Also, the methylation level of the *NEUROD1* gene was significantly lower on ecMYC cell lines (Fig. 6F). The presence of enhancers characterized by H3K27ac and H3K4me3 in the TSS of *NEUROD1* was discovered in the epigenomic landscape of the NCI-H2171 cell line with publicly available data (Fig. 6G). The *MYC* bound to the enhancer provides direct evidence for *MYC* regulation of *NUEROD1* by *MYC* (Fig. 6G). MED1 and RNA polymerase II signals show that *NEUROD1* transcription is active under the regulation of *MYC* (Fig. 6G). Together, these results indicate that ecMYC drives the expression pattern of the SCLC-N subtype in SCLC cell lines with ecMYC. Thus, we could relate the expression pattern of SCLC to genomic variation.

**Fig. 7:**
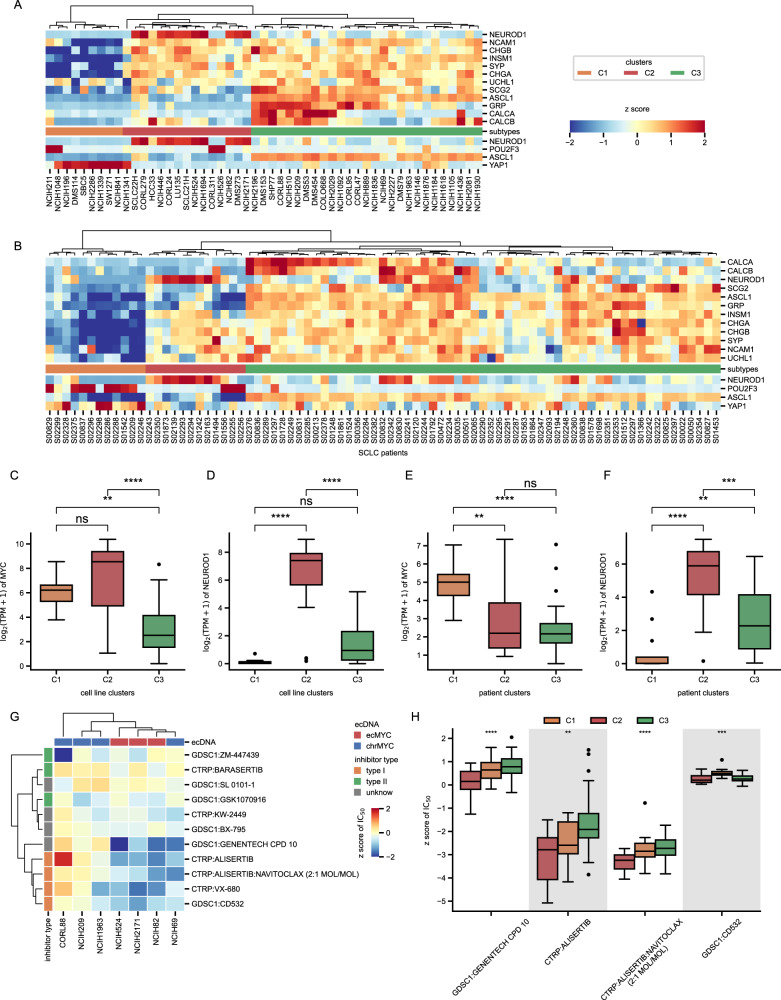
Expression patterns of SCLC subtypes. **A** Unsupervised hierarchical cluster analysis of neuroendocrine markers from human SCLC cell lines. **B** Unsupervised hierarchical cluster analysis of neuroendocrine markers from human SCLC patients. **C**, **D** The expression level of *MYC* and *NEUROD1* between SCLC cell line clusters. **E**, **F** The expression level of *MYC* and *NEUROD1* between SCLC patient clusters. **G** Heatmap of 7 SCLC cell line response to 11 AURK inhibitors. **H** AURKi response prediction between patient clusters.

In addition to these few cell lines of SCLC, we tried to extend the finding to more samples. Hierarchical clustering analysis based on neuroendocrine markers [30] showed that 51 SCLC cell lines were separated into 3 clusters, and all 3 ecMYC-containing cell lines were partitioned into cluster C2 (Fig. 7A) with high expression of *MYC* (Fig. 7C) and *NEUROD1* (Fig. 7D). We also observed a similar pattern of classification in patients (Fig. 7B). Samples with high levels of *NEUROD1* expression were partitioned to the C2 cluster (Fig. 7B, F), but unlike the cell lines, the C2 cluster had a lower overall expression of *MYC*. However, they had the highest *MYC* expression individual samples (Fig. 7E). This could be a sampling bias as most of these samples are limited-stage SCLC, and *MYC* amplification is relatively rare [30, 33, 34]. Interestingly, we observed that samples from the C1 cluster in either cell lines or patients had high *MYC* and *POU2F3* or *YAP1* expression (Fig. 7B), suggesting that there may be an association between them or *MYC* drives a greater number of subtypes than just SCLC-N. However, elevated *NEUROD1* expression has been shown to be a clear marker of poor prognosis. Conversely, increased expression of *POU2F3* is indeed a marker of good prognosis [35], so the two may have entirely different driving mechanisms. SCLC has been reported to have unique therapeutic vulnerabilities between subtypes, and SCLC-N is sensitive to Aurora kinase inhibitors (AURKi) [30, 34, 36]. AURKi has been shown to induce degradation of *myc* in hepatocellular carcinomas mice [37], so it is reasonable to hypothesize that AURKi is equally effective in SCLC driven by ecMYC. On the basis of drug screening data obtained from the Genomics of Drug Sensitivity in Cancer (GDSC) and Cancer Therapeutics Response Portal (CTRP) databases [38, 39], we observed similar results for cell lines with ecMYC and ecDNA containing *MYCN* that were sensitive to type I AURKi (Fig. 7G and Supplementary Fig. S3F). This result further demonstrates the driving role of ecMYC in these three cell lines. On the other hand, cell lines with amplification of *MYCL* (Supplementary Fig. S3F), which is also a member of the *myc* gene family, are insensitive to AURKi (Fig. 7G). In addition, we also predicted the patient’s response to type I AURKi, and the results showed that the C2 cluster samples were the most sensitive (Fig. 7H). These results suggest that SCLC-N has unique therapeutic vulnerabilities and may be related to ecMYC.

## Discussion

Since its discovery half a century ago, ecDNA has gradually been recognized as a hallmark of cancer [1]. Non-chromosomal inheritance, which drives the amplification of oncogenes on ecDNA, allows tumors to rapidly evolve their genomes, gain a survival advantage and resist to drug treatment [1, 3, 9]. Existing ecDNA identification methods typically require complex steps of circular DNA enrichment library construction steps or the analysis of large amounts of data. For this reason, circlehunter pipeline, developed for identifying ecDNA using regular ATAC-Seq data, is a necessary complement. Analysis of both simulated and known samples shows that circlehunter has high accuracy in identifying complex circular structures from linear genomes. Our analysis of ATAC-Seq data from 547 tumor samples predicted 1312 ecDNAs in 94 samples. Data from CCLE showed that 37 oncogenes were amplified on ecDNAs, which is consistent with the essential characteristics of ecDNA. These results demonstrate that we could find cancer-associated ecDNA from existing regular ATAC-Seq data and interpret cancer characteristics from a different perspective. For example, ecMYCs were identified in three SCLC cell lines. Genomic and transcriptomic data from publicly available sources indicate that these three cell lines have essential characteristics of ecDNA, including oncogene copy number gain and increased expression levels. Further analysis showed that ecMYC drives these 3 cell lines to exhibit the same expression pattern characteristic of the SCLC-N subtype. Of these, epigenomic data from the NCI-H2171 cell line further supported a regulatory role of ecMYC on *NEUROD1*, an essential factor in the expression pattern of SCLC-N subtypes. Drug screening data showed that these cell lines were sensitive to AURKi, a potential class of drugs targeting *MYC* amplification is a promising drug for treating SCLC patients with ecMYC.

However, there are also disadvantages to using ATAC-Seq data for ecDNA identification. As the sequencing depth obtained by ATAC-Seq is not a direct reflection of the copy number of the original template, without this information, circlehunter can fail to construct circles that contain repeated or foldback segments, such as homogeneously staining region (HSR) and breakage fusion bridge (BFB) cycle. In addition, other complex structural variants that are detectable by bioinformatic tools such as JaBbA [40] or LINX [41], which utilize depth-balanced junction graphs, may not be identified by circlehunter without this information. Therefore, it is important to note that the current benchmarking strategy has limitations, and we cannot guarantee the accuracy of ecDNA calls for all types of focal amplifications. Future studies should explore the rate at which this happens and devise strategies to improve the accuracy of ecDNA detection for other types of focal amplifications. It has been reported in the literature that copy number profile can be predicted using single-cell ATAC-Seq data [42], and the addition of the copy number profile can allow circlehunter to remove some of the computational sub-structure by calculating the copy number balance on either side of the breakpoint. However, the application of circlehunter to single-cell ATAC-Seq data has yet to be explored. At the same time, short read-length sequencing has a natural disadvantage for detecting structural variants, which may lead circlehunter to discover more computational sub-structures, as in the case of the COLO320DM cell line. But no single solution is perfect, and in our scenario, if the copy number of ecDNA has not yet increased to a level at which it can be identified early in the amplification process, this ecDNA becomes difficult to identify using regular WGS data, for example, the AmpliconArchitect software, which requires copy number variation as input. We believe that the circlehunter should still be valid in this instance, given that ecDNA lacks nucleosome wrapping and will yield more library fragments generated by Tn5 cutting, which can be considered a means of enrichment to some degree. We also note that most of the samples in the dataset we analyzed were cell lines, and the proportion of individual cancer types deviated significantly from each other. Additional data are needed to improve our understanding of the possible issues with using ATAC-Seq for ecDNA analysis. As circlehunter is built on the basis that ecDNA has less higher-order compaction than chromosomes. This finding has been reported in large-scale and empirical studies [6, 9], but more data are needed to support whether all ecDNAs share this characteristic. On the other hand, if other classes of focal amplification have misregulated chromatin, they may be detected as false-positive ecDNA by circlehunter. In closing, we provide a tool to explore cancer-associated ecDNA, utilizing new or existing data, and offer a unique entry point for oncology research.

## Materials and methods

### The circlehunter pipeline

The circlehunter pipeline was constructed using snakemake [43]. The main steps are as follows (Supplementary Fig. S5).

### Preprocess

Input FASTQ files were processed by fastp [44] to remove adapter and low-quality bases. Clean reads were mapped to the genome using bwa-mem [45]. Duplicated reads were marked by samblaster [46] and then transformed into coordinate sorted BAM files using samtools [47]. Reads mapped out of ENCODE blacklist [48] with a MAPQ > 10 and not marked as duplicates will be kept and used for the next steps.

### Enrich region identify

For consecutive enrich regions, all reads were used as input to call peak using MACS2 [49] with parameters --nomodel --nolambda -p 0.05. This implies that all reads will be pileup as depth of each base. The *P* value for each base will then be determined by a Poisson distribution using each base’s depth, with the average depth of the genome as *λ*. Sequential bases with a *P* value < 0.05 will be considered as significant peaks. Peaks with distances less than 12.5 kb will be merged as one consecutive enrich region using bedtools [50].

For discordant reads pair enrich regions, discordant read pairs (insert size >1500 bp as default but can be adjusted) were extracted and pileup. Their ratio to the depth of the same base was then calculated. Using a Poisson distribution with the mean ratio of the whole genome as *λ*, the ratio of each base will be computed as a *P* value, similar to consecutive enrich region identification. Consecutive bases with *P* value < 0.05 will stitch as discordant enrich regions. These steps were performed by MACS2 bdgcmp with parameter -m ppois and bdgpeakcall with parameter -c 1.301 $( = - \log _{10}0.05)$. Only regions that overlap with consecutive enriched regions are kept.

### Construct the breakpoint graph

The breakpoints graph relies on MultiGraph in NetworkX [51]. All discordant read pair enrich regions are added as nodes. Two types of edges are then added according to enriched regions. Two discordant enrich regions share paired-end reads with the same reads ID, which means a discordant type edge. The user can specify a minimum number of reads IDs with the same mapped orientation that must be present for a discordant edge to be formed. The default setting is the inverse survival of the Poisson model used for enrichment assessment. When a discordant type of edge is added to the graph, the orientation of the reads that support the edge is recorded in both nodes it connects. This direction is used as the extension direction of this node. All nodes properly oriented to one another within one consecutive enrich region will be connected by consecutive type edges.

### Breakpoint estimate

Despite the discordant read pair enrichment region suggesting that a breakpoint is present, it does not provide the precise position of the breakpoint. Hence, we propose a Bayesian model to estimate a more precise breakpoint position. The breakpoint position is estimated by combining two distinct Bayesian models. Circlehunter takes discordant read pairs mapped in the discordant enrich region, because the reads stopping before the breakpoint, so it can only be used to estimate the minimum extended position of the breakpoint. Similar to the German tank problem [52], with the innermost base of the discordant reads enriched region as the origin and the distance from each possible breakpoint to the origin as *h*. The probability of observing reads with an end position less than *h* appearing in *L* follows a uniform distribution between 1 and *h*.

$$P_L\left( {L|h} \right) = \left\{ {\begin{array}{*{20}{l}} {0,} \hfill & {{{{\mathrm{if}}}}\,L > h} \hfill \\ {Uniform\left( {1,h} \right),} \hfill & {{{{\mathrm{otherwise}}}}} \hfill \end{array}} \right.$$

The other model is a simple normal distribution, where the reads aligned to the breakpoints will be clipped during the alignment process. Their alignment end position *S* should fall within a small ±*e* distance from the actual breakpoint. Thus, the probability of such reads’ alignment end positions occurring in *S* is observed to obey a normal distribution with *h* as the expectation and *e*^2^ as the variance.

$$P_S\left( {S|h} \right) = N\left( {h,e^2} \right)$$

Assuming that the probabilities of being observed for these two types of reads are independent, the two model posterior probabilities can be integrated as

$$\log P\left( {h|L_{1:n},S_{1:m}} \right) = \chi + \mathop {\sum}\limits_{i = 1}^n {\log } P_L\left( {L_i|h} \right) + \mathop {\sum}\limits_{i = 1}^n {\log } P_S\left( {S_i|h} \right)$$

where *χ* is the normalized parameters of the model, the maximum-likelihood estimates $\widehat h$ and 95% confidence interval are then obtained from the model through a grid search. The breakpoints estimated by the model will be applied to the breakpoint graph nodes.

### Search ecDNA from the breakpoint graph

The proper ecDNA will be reconstructed from the graph by a modified depth-first search algorithm. Since the breakpoint graph constructed by circlehunter comprises two types of edges connecting different breakpoints, a proper ecDNA circle needs to search along a path with two distinct types of edges. When searching for the next breakpoint by depth-first in the graph, the type of the edge connecting the next breakpoint must not be the same as the type of the edge connecting the prior breakpoint. Circlehunter will first search for segments with greater size and higher local depth to include more genes and to find more reliable ecDNAs. All possible circles and breakpoint confidential intervals will be output in BED format as distinct candidate ecDNA structures. Typically, circlehunter will cover all nodes. However, a minimal number of samples that undergo complex rearrangement will result in many alternative sub-structures, and the user can opt to restrict the output (default as 1000).

### Accuracy of circlehunter

We randomly generated 500 ecDNAs in order to evaluate the accuracy of circlehunter and to compare it to the existing circle_finder approach that can analyze ecDNA from ATAC-Seq data. The sizes of fragments vary from 5 kb to 10 Mb, and the number of fragments in a single ecDNA varies from 1 to 50, covering a wide range of ecDNA conditions. All segments were randomly selected from the GRCh38 not N regions. Each segment is randomly assigned a ligation direction, and sequences are extracted from the genome accordingly. All sequences from the same ecDNA were sequentially ligated into one large circular sequence. Subsequently, pair-end sequencing simulation was performed using art employing the Illumina default profile [53]. The ATAC-Seq data of the GM12878 sample (GSE170245) from ENCODE is blended as background with the output of 20 ecDNA sequencing simulations for each run. This will create a sample containing 20 randomly generated ecDNAs, and 25 such samples will be produced in total, resulting in 500 ecDNAs. The simulation method for one-segment ecDNA is the same as that for multi-segment ecDNA except for limiting the number of fragments per ecDNA. ATAC-Seq data for three samples with known ecDNA were downloaded from GEO (Supplementary Table S2), with two biological replicates per sample. These samples were analyzed based on the hg19 references.

### Identify ecDNA in historical data

ATAC-Seq data for 547 tumor samples were obtained from the GEO database (Supplementary Table S3). Manual curation was conducted on the search results for the term “ATAC-Seq”. Only samples that were identified as being derived from cancer and had not received any special treatment, such as drug or gene modifications, were kept. The FASTQ format raw data were obtained from Sequence Read Archive (SRA) [54] using SRA Toolkit. And the analysis was conducted with circlehunter with the hg38 genome as the reference using default settings. Initial results were filtered according to the following conditions: fold enrich >10, discordant reads proof a junction >2, less than 5% of length overlap with RepeatMasker [55] repeat regions. Gene annotations were downloaded from UCSC refSeq (refGene) [56]. AllOnco (http://www.bushmanlab.org/links/genelists) cancer gene list curated by Bushman Lab was used as an oncogene list. Copy number, expression level, and gene effect data for cancer cell lines sourced from CCLE [26] can be accessed from DepMap (https://depmap.org/portal/). Clinical copy number and coding driver mutations from PCAWG were downloaded from Xena [57].

### Identify ecDNA in SCLC

Nineteen ATAC-Seq data from 8 SCLC cell lines (Table S4) were downloaded from the GEO database and analyzed using hg38 as the reference. Data for copy number and expression level of the gene were obtained from DepMap Public 22Q1. GSEA and GO analyses were performed using GSEAPY (https://github.com/zqfang/GSEApy), and the gene sets were downloaded from MSigDB [58]. Differently expressed genes were identified using the Student *t* test. Differentially expressed genes between the ecMYC group and chrMYC were defined as the absolute value of log foldchange >2 and *P* value < 0.05. Transcriptome sequencing data of human SCLC tumor samples (*n* = 81) were obtained from published literature [33], and SCLC cell lines (*n* = 51) were obtained from CCLE. The log2-FPKM cut-off to distinguish patients as having *MYC* high and *MYC* low is the upper quartile for all patients. The same cut-off value is used to classify cell lines. Epigenomic sequencing data for the NCI-H2171 cell line were downloaded from GEO with accession GSE36354, aligned to the hg38 reference using bwa-mem, pileup as per million reads signal and scaling to the smallest sample using MACS2. Drug screening data were sourced from GDSC [38] and CTRP [39] and downloaded from DepMap. We used drug response data from oncoPredict’s [59] prediction given the large number of missing data in the original screening data. The model was trained using all accessible SCLC screening data and was later used to predict the response of 51 cell lines to 11 AURKi drugs. The response of SCLC patients to AURKi was similarly predicted by oncoPredict.

### Statistical analysis

The statistical methods are described in the corresponding statements and figure legends. Statistical analysis is performed by SciPy or Pingouin in Python.

## Supplementary information

Supplementary lengend

Fig. S1

Fig. S2

Fig. S3

Fig. S4

Fig. S5

Table S1

Table S2

Table S3

Table S4

Table S5

## Data Availability

This study did not generate new data. All publicly available data used are already listed in “Materials and methods” or Supplementary materials.

## Code availability

The source code of circlehunter, including the simulation pipeline and configuration, is available at https://github.com/suda-huanglab/circlehunter.
