# Supplementary material for: Circlehunter: a tool to identify extrachromosomal circular DNA from ATAC-Seq data
Source: Oncogenesis. 2023 May 22;12(1):28. doi: 10.1038/s41389-023-00476-0 (PMC10202962; doi:10.1038/s41389-023-00476-0)
Supplement: Supplementary file 1 — Supplementary lengend [file 41389_2023_476_MOESM1_ESM.docx]

supplementary legends

**Fig S1. Simulation test statistics.**

(A) The ecDNA detection result of GSE118092 between circlehunter and circle_finder. Circlehunter accurately identifies two deletions in the ecDNA by calculating whether the regions are consecutively enriched. Evidence read coverage is in log scale. (B) Evidence read used by circlehunter and circle_finder to support the junction between chr7:55019017-55211628. (C) Length distribution of simulation ecDNAs. (D) Percent distribution of the number of segments of individual simulation ecDNA. (E-F) Precision and recall of multi segments ecDNA detection with varying mock ecDNA local depth at 100 bp read length. (G-H) Precision and recall of multi segments ecDNA with varying read length at 30× local depth. Circlehunter-s: A true-positive result is considered when the circlehunter result covers 95% of the simulated ecDNA and has the same ligation structure; Circlehunter-b: A true-positive result is considered when the circlehunter result breakpoint confidential intervals cover simulated ecDNA breakpoint and have the same ligation structure; Circlefinder-s: A true-positive result is considered when the Circle_finder result covers 95% of the simulated ecDNA. (I-K) Wall time, memory usage, and IO traffic with varying overall depth downsampled using samtools from GSE118092. Error bars indicate s.d. The performance tests were conducted on a computer with an AMD Ryzen Threadripper 1950X CPU and 100GB of RAM. The results represent the resources required for a single-core runtime, excluding the preprocessing part.

**Fig S2. Metrics of historical samples.**

(A) Sample type distribution of historical samples. (B) Primary disease distribution of historical samples. (C) Average sequencing depth for historical samples. (D) Read length for historical samples. (E) Fraction of ecDNA present in primary and metastatic samples.

**Fig S3. Genes amplified on ecDNA.**

(A) Kernel density estimate of copy number ascending rank between samples within ecDNA containing the gene and samples without ecDNA containing the gene. (B) Kernel density estimate of expression level ascending rank between samples within ecDNA contained the gene and samples without ecDNA contained the gene. (C) Kernel density estimate of gene effect rank between samples within ecDNA contained the gene and sample without ecDNA contained the oncogene. (D) Correlation between gene copy number fold change and expression level fold change, fold change is calculated as the copy number or TPM of the gene between the median of copy number or TPM of the gene within same cancer. Red for samples in which ecDNA containing the gene appears, and gray for samples in which ecDNA containing the gene was not detected. (E) *MYCN* contained ecDNA from NCI-H69. (F) The expression level of 3 *myc* family genes in 7 SCLC cell lines.

**Fig S4. SCLC-N expression pattern.**

(A) Differentially expressed gene between cell lines with ecMYC and chrMYC. (B) GSEA analysis between MYC-high and MYC-low SCLC patients with differentially expressed genes between ecMYC and chrMYC cell lines. (C) Gene effect of 3 SCLC cell lines. The curves show the kernel density estimation of the gene effect for all genes corresponding to the cell line. The boxes show the five-number summary of gene effect for all genes corresponding to the cell line, including the minimum score, lower quartile, median, upper quartile, and maximum score. The triangle indicates the ranking position of the gene effect of the corresponding gene for the cell line. (D) Correlation of expression level between *MYC* and *NEUROD1*.

**Fig S5. Workflow of circlehunter.**

**Table S1. Simulation test results**

**Table S2. Samples with Known ecDNA**

**Table S3. Historical samples used in this study**

**Table S4. SCLC samples used in this study**

**Table S5. SCLC expression pattern**
