# Supplementary figures and images for: Circlehunter: a tool to identify extrachromosomal circular DNA from ATAC-Seq data
Source: Oncogenesis. 2023 May 22;12(1):28. doi: 10.1038/s41389-023-00476-0 (PMC10202962; doi:10.1038/s41389-023-00476-0)

A

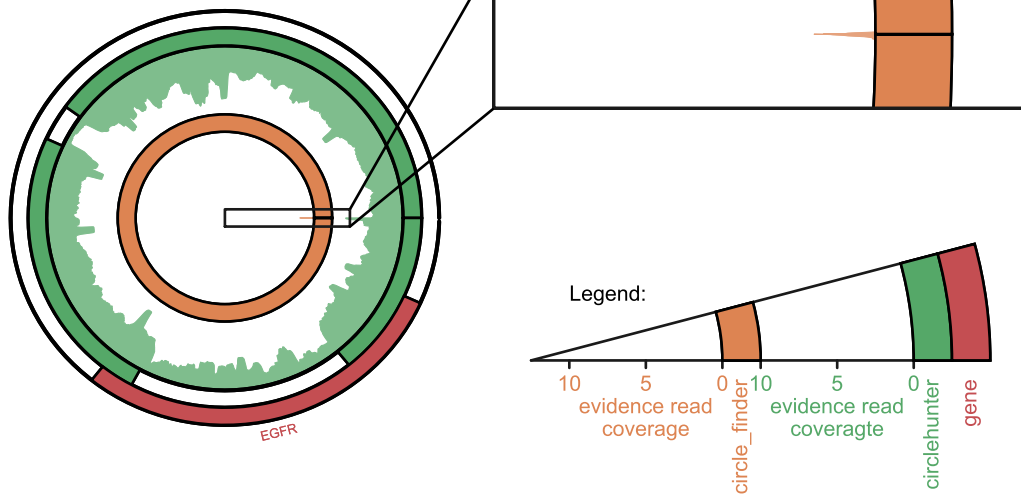

B

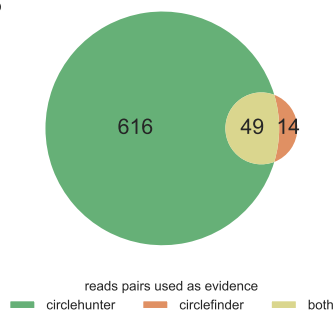

C

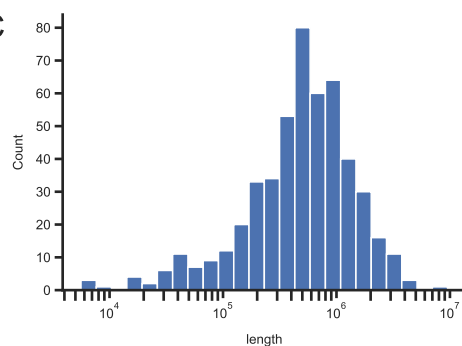

D

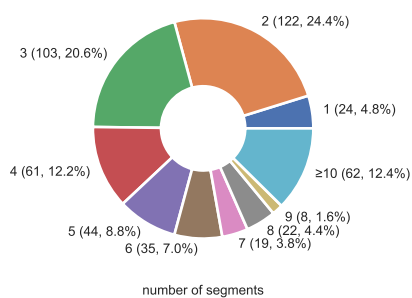

E

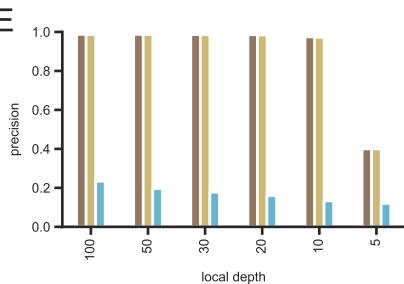

F

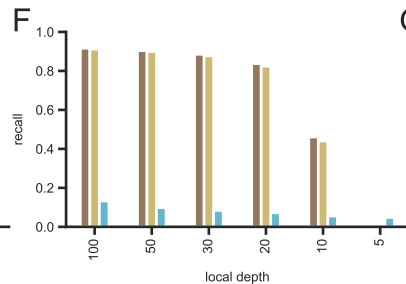

G

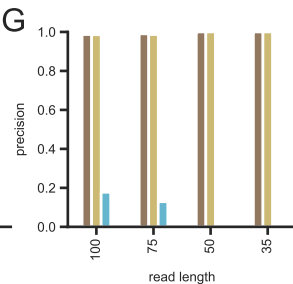

H

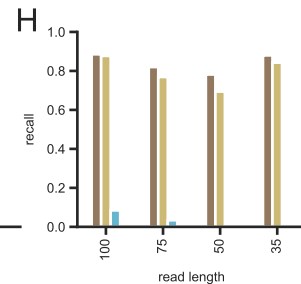

I

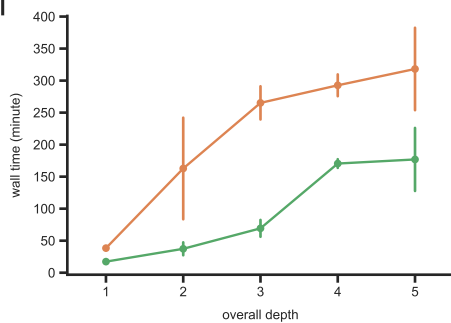

J

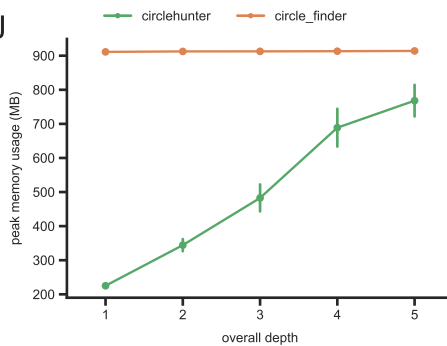

K

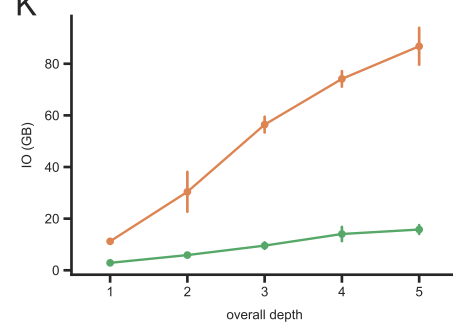

Supplement: Supplementary file 2 — Fig. S1 [file 41389_2023_476_MOESM2_ESM.pdf]

A

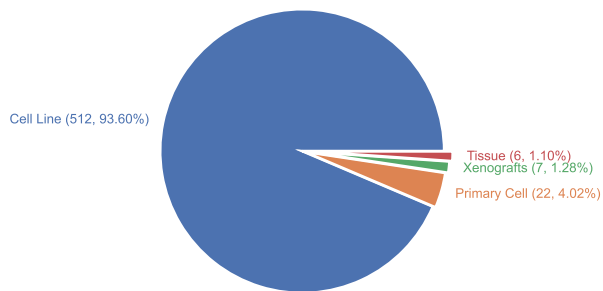

B

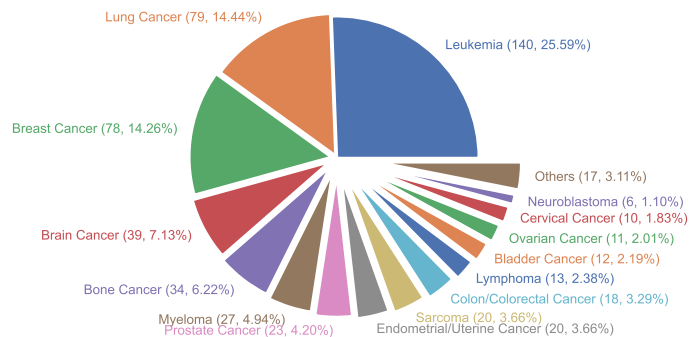

C

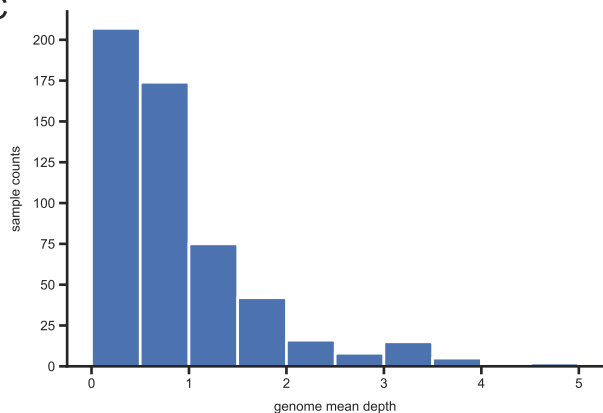

D

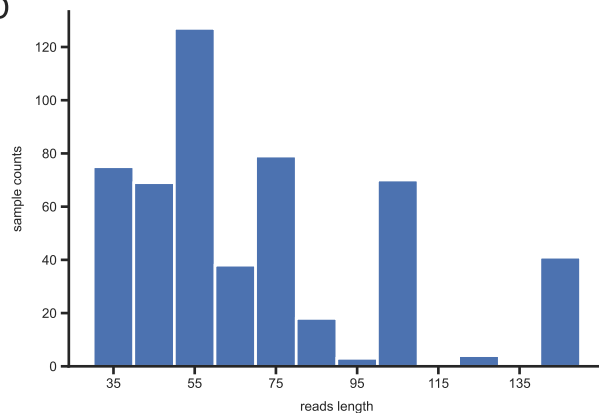

E

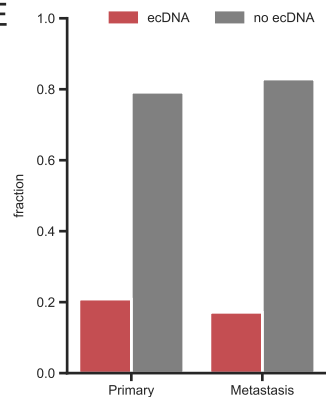

Supplement: Supplementary file 3 — Fig. S2 [file 41389_2023_476_MOESM3_ESM.pdf]

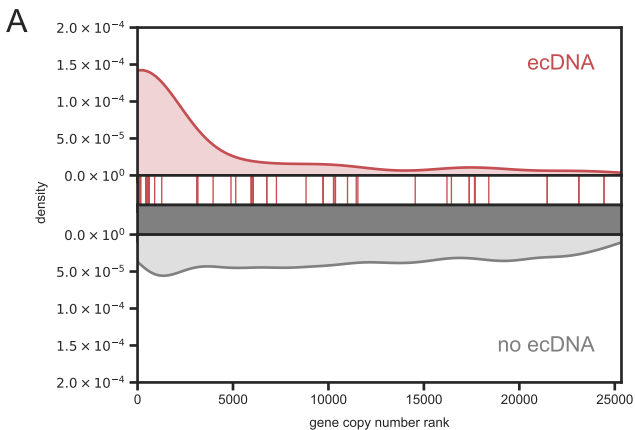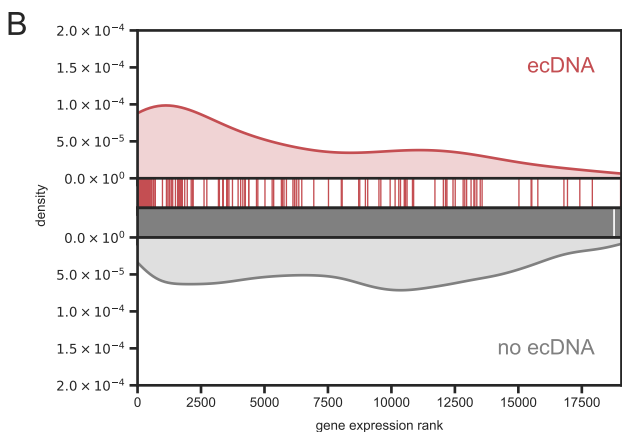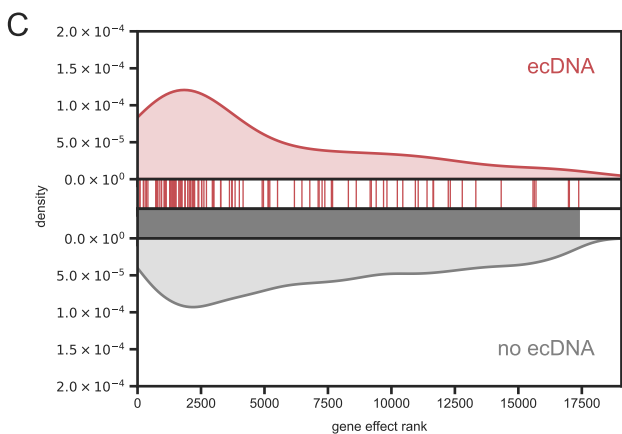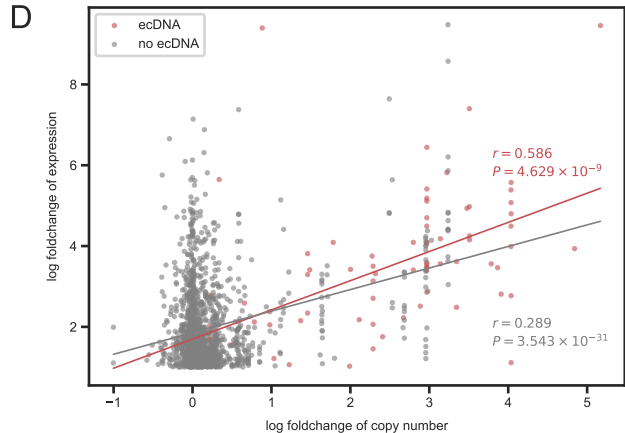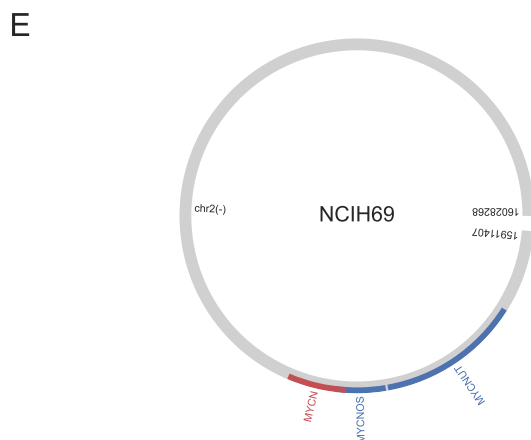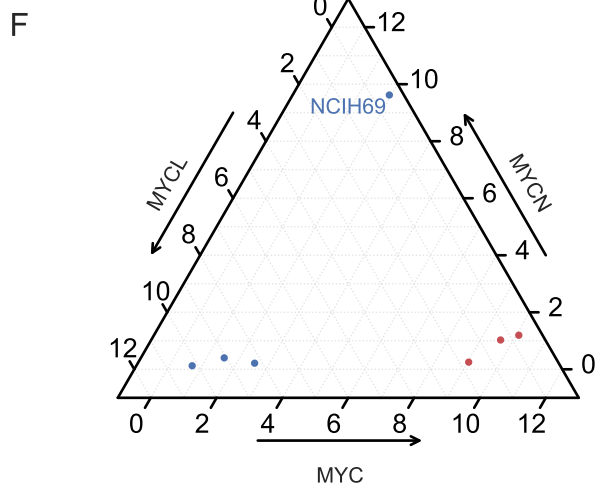

Supplement: Supplementary file 4 — Fig. S3 [file 41389_2023_476_MOESM4_ESM.pdf]

A

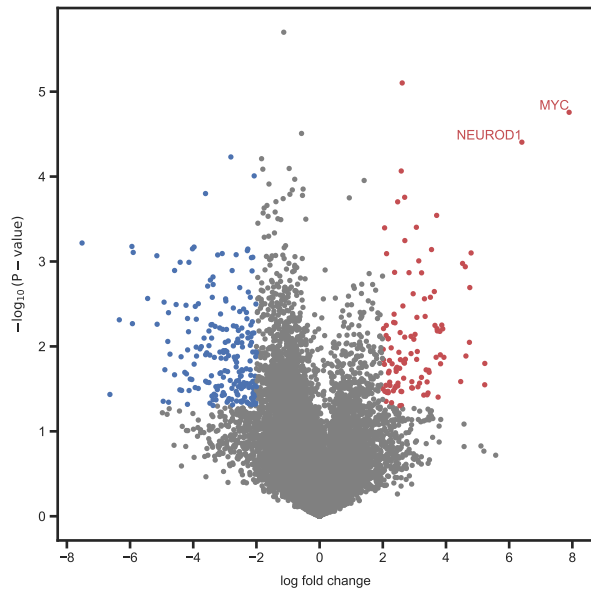

B

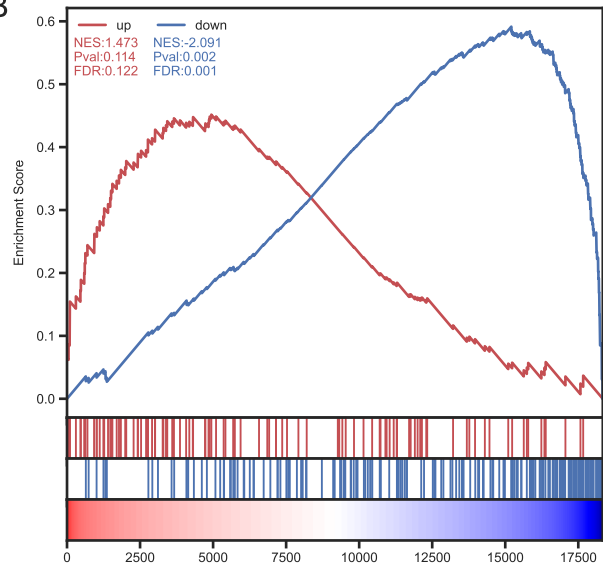

C

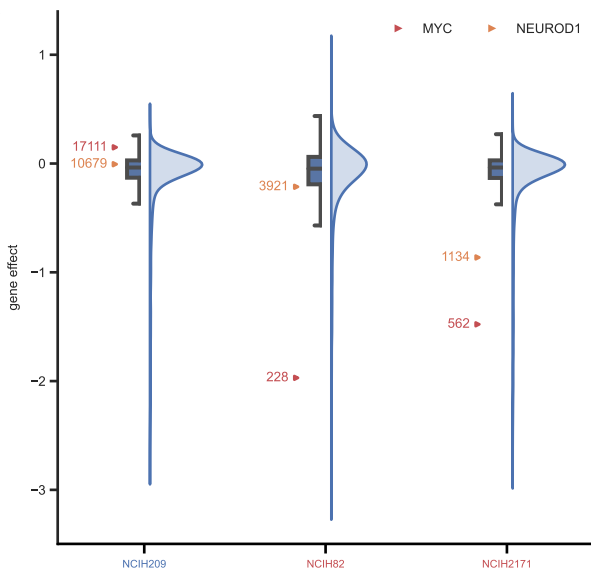

D

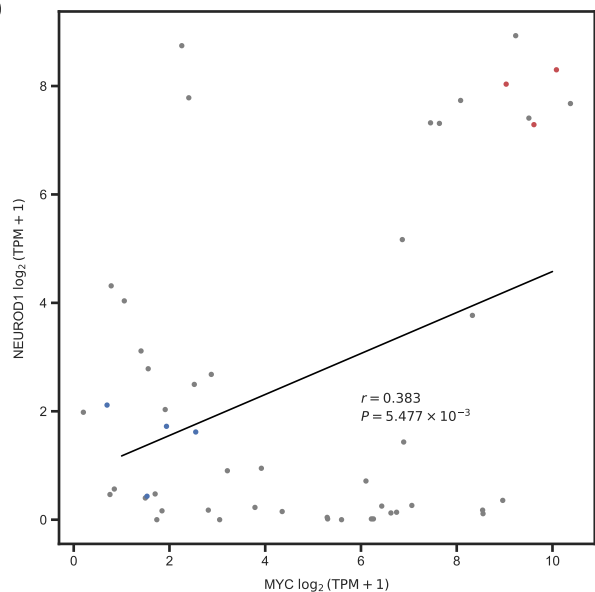

Supplement: Supplementary file 5 — Fig. S4 [file 41389_2023_476_MOESM5_ESM.pdf]

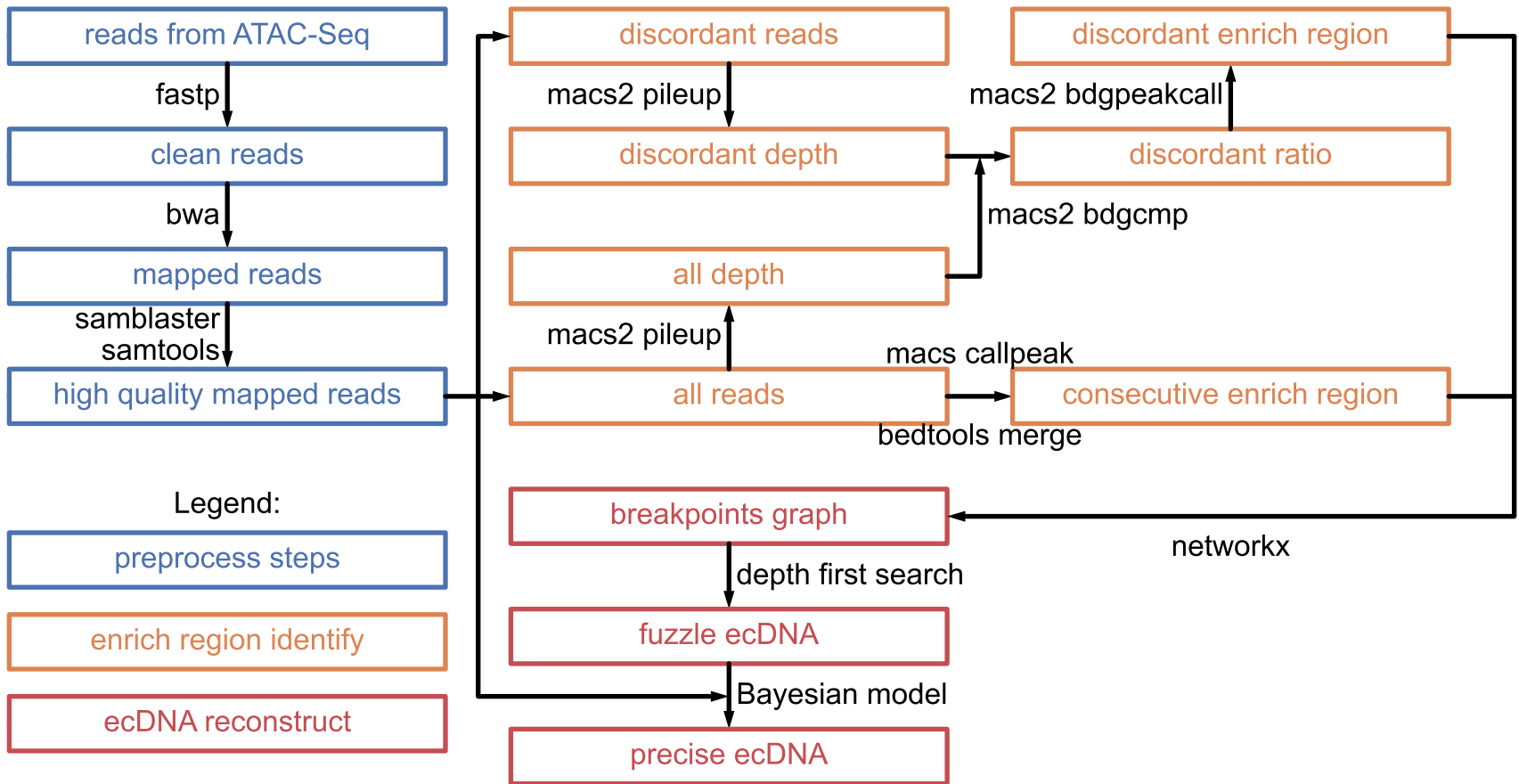

Supplement: Supplementary file 6 — Fig. S5 [file 41389_2023_476_MOESM6_ESM.pdf]
